# Artificial Intelligence and Digital Pathology for Molecular Classification of Endometrial Cancer

**DOI:** 10.3390/ijms27167341

**Published:** 2026-08-17

**Authors:** Yesul Jeong, Sungman Hong, Sangjeong Ahn, Sung Hak Lee

**Affiliations:** 1 Department of Hospital Pathology, St. Vincent’s Hospital, College of Medicine, The Catholic University of Korea, Seoul 06591, Republic of Korea; seulyool@catholic.ac.kr; 2 Department of Biomedical Informatics, College of Medicine, Korea University, Seoul 02841, Republic of Korea; sungmanhong@gmail.com; 3 Department of Pathology, College of Medicine, Korea University, Seoul 02841, Republic of Korea; 4 Department of Hospital Pathology, Seoul St. Mary’s Hospital, College of Medicine, The Catholic University of Korea, Seoul 06591, Republic of Korea

**Keywords:** endometrial cancer, molecular classification, mismatch repair deficiency, microsatellite instability, POLE mutation, tumour mutational burden, digital pathology, whole-slide imaging, artificial intelligence, deep learning

## Abstract

Endometrial cancer is one of the most rapidly increasing gynaecological malignancies worldwide. The clinically adapted molecular classification of endometrial carcinoma, derived from The Cancer Genome Atlas, comprises four major subtypes: POLE-mutated, mismatch repair-deficient, p53-abnormal expression, and no specific molecular profile. Its clinical implementation has improved prognostic stratification, risk assessment, and treatment decision-making in patients with endometrial carcinoma. However, current workflows rely on immunohistochemistry and targeted sequencing, which increase costs, turnaround times, and infrastructure requirements, thereby limiting their universal adoption in routine clinical practice. Recent advances in artificial intelligence (AI), particularly deep learning models capable of predicting molecular features directly from H&E-stained whole-slide images, have emerged as promising tools for precision oncology. In addition to reproducing established molecular classification, these approaches may reveal previously unrecognised biomarker-defined histologic patterns that are difficult to detect using conventional methods. This article synthesises the current evidence on AI-based molecular classification in endometrial carcinoma from a pathologist-centred perspective, emphasising the biological rationale, methodological limitations, and future directions for clinical translation.

## 1. Introduction

Endometrial cancer (EC) is the sixth most frequently diagnosed malignancy in women, accounting for 434,620 new cases and 100,680 deaths worldwide in 2024 [1]. The incidence of EC is particularly high in developed countries and is closely associated with established risk factors, including obesity, metabolic syndrome, ageing, and prolonged exposure to unopposed oestrogen [2,3].

In 2013, The Cancer Genome Atlas (TCGA) Research Network established a molecular classification for EC by integrating genomic, transcriptomic, and proteomic data [4]. This landmark study stratified EC into four molecular subgroups: POLE-ultramutated, microsatellite instability (MSI), copy number (CN)-low, and CN-high subgroups. Although this framework has transformed the biological understanding of EC, the sequencing-based and multi-omics approaches used in the original TCGA study are difficult to implement directly in routine diagnostic practice. Accordingly, subsequent translational efforts have focused on clinically feasible surrogate algorithms that preserve the biological and prognostic relevance of TCGA classification [5,6]. In this context, the Proactive Molecular Risk Classifier for Endometrial Cancer (ProMisE) and TransPORTEC groups proposed more practical approaches based on next-generation sequencing (NGS) to detect pathogenic POLE exonuclease domain mutations (POLEmut), immunohistochemistry (IHC) to identify mismatch repair deficiency (MMRd) and aberrant p53 expression (p53abn), and the classification of tumours lacking these alterations as no specific molecular profile (NSMP) [7,8].

This molecular classification has substantially improved EC management by refining prognostic assessment, resolving diagnostically ambiguous high-grade cases, guiding adjuvant treatment decisions, enabling biomarker-driven clinical trials, and improving diagnostic reproducibility by reducing the reliance on histology alone [9,10]. However, current workflows still rely on IHC, polymerase chain reaction (PCR)-based assays, and NGS. These methods require additional tissue, cost, turnaround time, laboratory infrastructure, and technical expertise, which may limit the scalable implementation of molecular classification, particularly in resource-limited settings.

Digital pathology provides a potential translational bridge between routine histopathology and molecular cancer care. The widespread adoption of slide scanners and whole-slide images (WSIs) has created a technical foundation for the application of artificial intelligence (AI) in routine pathology practice [11,12]. In particular, deep learning (DL) models have emerged as promising tools for inferring molecular features directly from haematoxylin and eosin (H&E)-stained WSIs [13]. These approaches may complement established molecular assays by identifying histomorphology patterns associated with molecular subtypes, prioritising cases for confirmatory testing, and supporting more timely and accessible molecular risk stratification.

**Figure 1:**
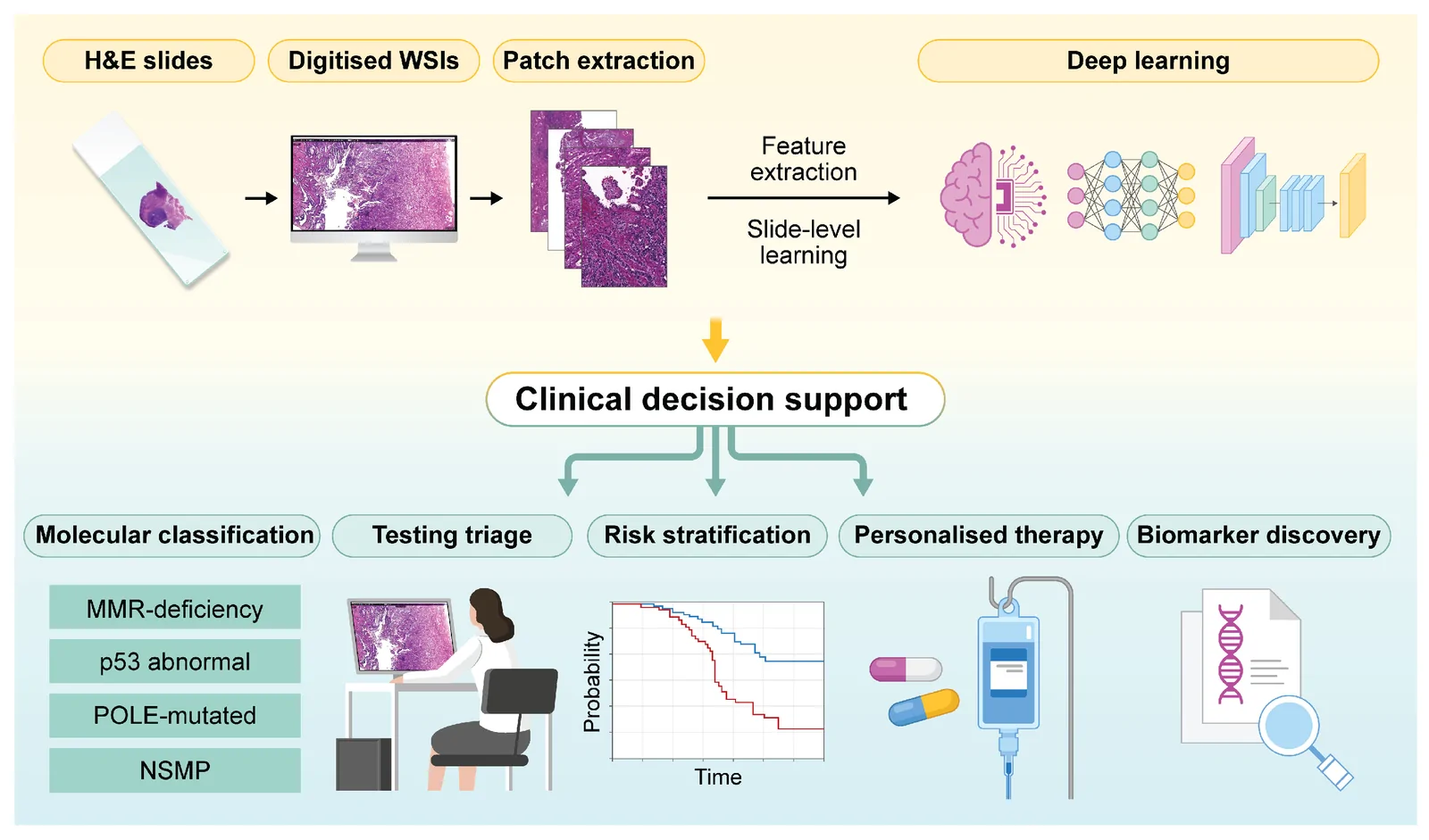
Workflow of AI-based molecular inference from H&E-stained WSIs for clinical decision support in endometrial cancer. Artificial intelligence-based analysis of routine H&E-stained whole-slide images can infer the molecular features of endometrial cancer and support clinical decision-making. By linking histomorphology with molecular subtype prediction, testing triage, risk stratification, personalised therapy, and biomarker discovery, digital pathology may serve as a translational bridge toward more accessible molecularly informed care. Black arrows indicate the computational image-processing workflow, yellow arrows indicate the transition to clinical decision support, and green arrows indicate downstream clinical applications. Abbreviations: MMRd, mismatch repair deficiency; NSMP, no specific molecular profile; p53abn, aberrant p53 expression; POLEmut, pathogenic POLE exonuclease domain mutations.

Several recent reviews have addressed the expanding role of AI and digital pathology in gynaecological oncology; however, many have taken a broad perspective across tumour types, imaging modalities, and computational tasks [14,15]. In contrast, our study aims to present how AI can learn relevant histologic features with specific molecular biomarkers from WSIs, and its interpretability and adoptability in real-world clinical workflows from the perspective of pathologists, rather than focusing on AI architecture technologies. In addition, we investigated how these approaches can be integrated into clinical pathology and oncology workflows to improve the prognosis of patients with EC (Figure 1).

We synthesised the current evidence on AI-based molecular inference in EC using digital pathology, with particular emphasis on four-class molecular subtype and tumour mutational burden (TMB) prediction, and critically evaluated the interpretability, cross-study heterogeneity, and requirements for safe clinical deployment.

## 2. Literature Search Strategy and Study Selection

**Figure 2:**
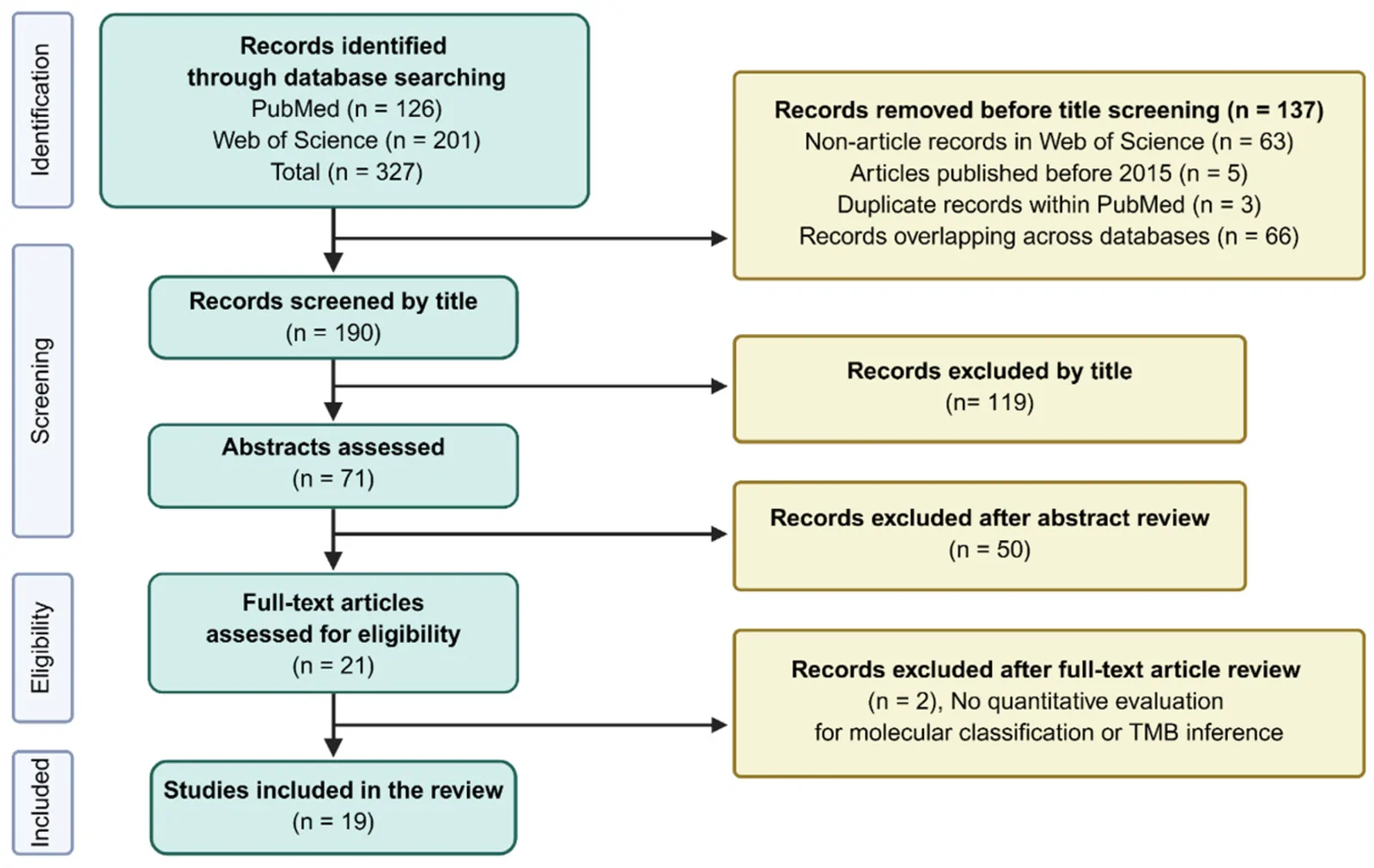
Flow diagram of literature identification and study selection. Created in BioRender. JEONG, Y. (2026) https://BioRender.com/39tok7x.

A structured literature search was conducted in PubMed and the Web of Science to identify original studies investigating AI-based prediction or inference of molecular subtypes and related molecular biomarkers, including TMB, in EC using routine H&E-stained histopathological images. The search covered publications from 1 January 2015 to 19 July 2026 (Figure 2).

The search strategy comprised three concept blocks: (1) EC; (2) AI, machine learning, DL, transfer learning, hierarchical learning, ensemble learning, multimodal DL, or foundation models; and (3) molecular classification or molecular biomarkers, including MMRd, MSI, POLEmut, p53/TP53 abnormality, CN alterations, NSMP, and TMB. Synonymous terms within each block were combined using the Boolean operator “OR,” and the three concept blocks were combined using “AND.” Medical Subject Headings and text-word terms were used for PubMed, whereas keyword-based topic searching was used for Web of Science. The complete database-specific search strategies are provided in Supplementary Table S1.

Studies were eligible if they met all of the following criteria: (1) original full-text research articles; (2) inclusion of patients or tissue specimens with EC; (3) analysis of routine H&E-stained histopathological images, including WSIs or tissue microarray images (TMAs); (4) application of AI, including CNN to multimodal and foundation deep learning methods; (5) prediction of an established EC molecular subtype or related molecular biomarkers as predefined targets, including POLEmut, MMRd/MSI, p53abn/TP53 alterations, CN-high or -low groups, NSMP, and TMB; and (6) presentation of quantitative evaluation results of molecular prediction performance.

Reviews, systematic reviews, meta-analyses, editorials, comments, letters, and guidelines were excluded. Studies were also excluded if they (1) did not include EC; (2) did not use routine histopathological images, for example, used only genomic, transcriptomic, radiological, immunohistochemical, or spatial-omics data; (3) used image-derived molecular information as input data to predict prognosis or risk stratification; or (4) applied AI exclusively to tumour detection, segmentation, histological grading, diagnosis, prognosis, recurrence prediction, treatment response, or other outcomes.

The PubMed search yielded 126 records. After the removal of three duplicate records, 123 records underwent title screening. Of these, 68 were excluded based on their title. The abstracts of the remaining 55 records were reviewed, and 35 were excluded after the abstract assessment. The full texts of the remaining 20 articles were assessed for eligibility. Two articles were excluded: one used self-supervised cell representation learning for exploratory clustering of molecular subtype-associated histomorphology patterns without developing or quantitatively evaluating a patient- or slide-level molecular prediction model [16], and the other combined H&E-derived histology, image-derived molecular class prediction results, and anatomical stage data to predict postoperative distant recurrence rather than a molecular subtype or biomarker [17]. Consequently, 18 studies identified through PubMed were included in this review.

The Web of Science search yielded 201 records. Restricting the document type to “Article” reduced the number to 138, and five articles published before 2015 were excluded. After the removal of 66 records that overlapped with the PubMed results, 67 records underwent title screening. Of these, 51 were excluded based on their title. The abstracts of the remaining 16 records were assessed, and 15 were excluded after the abstract review. The full text of the remaining article was assessed and met the predefined eligibility criteria.

Overall, 190 unique records underwent title screening, 71 abstracts were assessed, and 21 full-text articles were evaluated for eligibility. Two articles were excluded after full-text review. The remaining 19 studies met the predefined eligibility criteria and were included in the narrative synthesis (Table 1). The major methodological strengths and principal limitations of the studies are summarised in Appendix A, Table A1. To aid interpretation, Table 2 defines common computational pathology terms, whereas Table 3 explains the key performance metrics and summarises the minimum reporting requirements for operating thresholds.

## 3. AI-Based Prediction of Molecular Subtypes in EC

Among the 19 studies included in this review (Table 1), seven focused exclusively on MMRd/MSI, while 17 (89%) evaluated MMRd/MSI as either the primary target or as part of a broader molecular classification. Seven studies (37%) investigated all four molecular subtypes (4-class ProMisE), three included TMB prediction (16%), and one specifically examined the relationship between NSMP and p53abn phenotypes. MMRd/MSI-related analyses were included in 89% of the selected studies, whereas none of the studies investigated POLEmut, p53abn, or NSMP as standalone prediction targets. These findings indicate that the evidence base for AI-based MMRd/MSI prediction is comparatively mature, whereas research on other molecular subtypes of endometrial cancer remains substantially underdeveloped.

The direct four-class molecular classification from H&E-based WSIs was first evaluated at scale with im4MEC and subsequently extended using multicentre CNN pipelines, hierarchical foundation models, interpretable end-to-end networks, and real-world foundation-model benchmarks [19,28,29,34,35,36]. An early proof-of-concept study evaluated four TCGA subtypes using separate one-versus-rest binary models [18]. This is not equivalent to a direct four-class classification: each binary model asks only whether one subtype is present, whereas a four-class model must distinguish all four alternatives simultaneously and assign the final subtypes. im4MEC achieved a macro-AUROC of 0.876 in the independent PORTEC-3 cohort [18,19], whereas hi-UNI achieved 0.879 in five-fold cross-validation without independent external validation [29]. An interpretable end-to-end model reported an external macro-AUROC of 0.844 in TCGA [36], while a multicentre CNN pipeline reported external AUROCs of 0.94 and 0.97 in two relatively small cohorts of 35 and 83 patients, respectively [28]. In larger real-world cohorts, the performance was lower, with an external macro-AUROC of 0.780 [34] and subtype-specific AUROCs ranging from 0.646 to 0.844 [35]. Across studies, p53abn was generally the most consistently discriminated subtype, whereas morphological overlap between POLEmut and MMRd/MSI and confusion between MMRd/MSI and NSMP were recurrent error patterns [19,34,35]. Because the cohort composition, reference assays, and validation designs differed, these results should not be interpreted as direct head-to-head comparisons.

Histology-based AI for molecular prediction does not directly detect DNA/RNA alterations or functional loss of proteins. Rather, it infers the molecular status from downstream morphological changes in tumour cells, tumour architecture, and tumour microenvironment (TME). Because of the strength and specificity of these morpho-molecular signals across molecular subtypes, their biological basis should be considered along with model performance, external validation, and clinical consequences of prediction errors (Table 4).

### 3.1. MMRd/MSI

#### 3.1.1. Biological and Clinical Significance of MMRd/MSI Type

MMRd/MSI tumours account for approximately 25–30% of ECs [37]. The DNA mismatch repair (MMR) system repairs single-base mismatches and short insertion–deletion loops that arise during DNA replication. Loss of MMR function leads to the accumulation of insertion–deletion mutations, particularly in repetitive microsatellite sequences, resulting in MSI. In routine practice, MMRd is assessed by IHC for MLH1, PMS2, MSH2, and MSH6 proteins, whereas MSI is detected using PCR- or NGS-based assays. Hypermutated MMRd/MSI tumours have an increased neoantigen load and frequently exhibit immune-rich histological features, providing a biological rationale for their sensitivity to immune checkpoint inhibitors. Accordingly, MMRd/MSI status is clinically important for both immunotherapy eligibility and Lynch syndrome screening [38,39,40].

#### 3.1.2. Morpho-Molecular Correlates of MMRd/MSI Type

MMRd/MSI tumours frequently exhibit prominent intraepithelial and stromal lymphocytic infiltration, inflammatory changes at the tumour–stromal interface, and variable glandular or solid growth. The model does not directly recognise MMR protein loss or microsatellite alterations; it primarily learns their downstream morphological consequences. This mechanism explains the substantial overlap between MMRd/MSI and POLEmut tumours, which similarly exhibit a high mutation burden and immune-rich morphology [19,28,36].

#### 3.1.3. AI Performance of MMRd/MSI Type

Among molecular targets, MMRd has emerged as the most extensively studied molecular subtype for AI-based prediction of H&E-stained WSIs in EC [20,21,22,23,26,32,33]. This is largely attributable to the availability of annotated ground-truth datasets generated by routine clinical assessment of MMRd/MSI status and the immunotherapeutic relevance of MMRd/MSI. Moreover, MMRd/MSI inference in EC benefits from a mature research ecosystem that has already been established for colorectal and gastric cancers [41,42].

Several studies have reported internal AUROCs of approximately 0.80–0.91 [20,22,23]. In direct four-class classification, im4MEC achieved an MMRd AUROC of 0.844 in the independent PORTEC-3 cohort [19]. The MMRNet achieved an internal AUROC of 0.897 and external AUROCs of 0.790, 0.807, and 0.863 across three independent datasets [26]. In contrast, later real-world four-class studies reported external AUROCs of 0.700 and 0.759, indicating that performance remains sensitive to cohort composition, laboratory ground truth, and domain shifts [34,35]. These estimates should not be directly ranked because the studies used different assays, specimen compositions, and experimental validation designs.

#### 3.1.4. Interpretability of MMRd/MSI Type

Subsequent studies have incorporated multi-resolution or hierarchical modelling strategies to analyse morphology across multiple histological scales. Whangbo et al. [22] proposed a multi-resolution ensemble model that integrates features from 2.5×, 5×, and 10× magnifications, thereby capturing both the broad architectural context and fine cytological details. Wang et al. [27] further extended this hierarchical approach by developing an IMAN that represents histology across the slide, bag, patch, and cell levels of the image. Studies suggest that multi-scale and multi-level attention modelling may enhance molecular inference from histopathology by integrating information distributed across tissue architecture, local image patches, and cellular-level features.

#### 3.1.5. Major Limitations and Clinical Implications of MMRd/MSI Type

The principal limitations are the non-equivalence among IHC-, PCR-, and NGS-derived labels [43,44], morphological overlap with POLEmut, subclonal MMR protein loss, and reduced generalisability across institutions and specimen types. The most plausible near-term application could be high-sensitivity triage or prioritisation for confirmatory MMR IHC or MSI testing.

### 3.2. p53abn

#### 3.2.1. Biological and Clinical Significance of p53abn Type

The p53abn subtype is characterised by TP53 alterations and CN-high, representing the most aggressive molecular category of EC. It accounts for approximately 5–15% of ECs and is consistently associated with poor clinical outcomes, supporting the need for intensified adjuvant treatment in appropriate settings [37,45]. In routine practice, p53 IHC is widely used as a practical surrogate method for assessing the p53abn status. Aberrant p53 expression patterns include diffuse, strong nuclear overexpression, complete absence of nuclear staining, and cytoplasmic expression [46,47,48].

#### 3.2.2. Morpho-Molecular Correlates of p53abn Type

p53abn tumours frequently show marked nuclear atypia, pleomorphism, hyperchromatism, a high nuclear-to-cytoplasmic ratio, and complex papillary, micropapillary, or serous-like architecture. They may also exhibit a high tumour-to-stroma ratio and relatively limited lymphocytic infiltration [19,36]. The recurrent association between p53abn status and high-grade histological features may provide a comparatively strong H&E signal. This explains why p53abn tumours are better predicted than other molecular subtypes, whose visible phenotypes are indirect or nonspecific.

#### 3.2.3. AI Performance of p53abn Type

Although dedicated p53abn-only studies are fewer than MMRd/MSI studies, p53abn generally shows the strongest subtype-specific discrimination in the direct four-class model. The im4MEC model achieved an AUROC of 0.928 in the independent PORTEC-3 cohort [19]. Guo et al. [36] reported external AUROCs of 0.950 in TCGA and 0.862 in the Suzhou cohort. Real-world foundation model studies reported external AUROCs of 0.844 and 0.851 and identified p53abn as the best-performing subtype in those cohorts [34,35].

#### 3.2.4. Interpretability of p53abn Type

Attention maps and high-scoring patches consistently highlighted nuclear pleomorphism, enlarged hyperchromatic nuclei, high-grade cytology, and papillary or serous-like architectures [19,28,36]. Single-cell analyses further showed that p53abn tumours had larger nuclei and high pleomorphism.

#### 3.2.5. Major Limitations and Clinical Implications of p53abn Type

Although p53 IHC results are highly concordant with TP53 mutation status, IHC-defined p53abn and sequencing-defined TP53 alterations are not fully interchangeable [48,49,50]. Discordance can arise from technical variations, interpretative differences between observers, sampling bias, and subclonal TP53 alterations resulting in heterogeneous p53 expression [46,51,52]. The most appropriate near-term application is a high-specificity rule-in flag that prompts confirmatory p53 IHC or TP53 testing, particularly in morphologically ambiguous tumours. However, AI-based p53abn prediction should not be used to independently determine high-risk classification or treatment escalation.

### 3.3. POLEmut

#### 3.3.1. Biological and Clinical Significance of POLEmut Type

The POLEmut subtype represents the most favourable prognostic category of EC, accounting for approximately 5–15% of cases [37]. Pathogenic POLE exonuclease domain mutations impair DNA replication proofreading and generate an ultramutated genomic phenotype characterised by an exceptionally high single-nucleotide variant burden [53]. Despite their favourable clinical behaviour, POLEmut ECs often exhibit aggressive histological features [37]. Accurate identification of POLEmut status is clinically important because current guidelines support adjuvant therapy de-escalation in patients with POLEmut EC [10,45]. However, definitive confirmation of POLE type requires targeted sequencing, which may not be universally accessible in all laboratories. Accordingly, AI-based prediction of POLEmut status from WSIs has been explored as a promising strategy to address the challenges associated with sequencing techniques.

#### 3.3.2. Morpho-Molecular Correlates of POLEmut Type

Morphologically, these tumours frequently retain endometrioid differentiation but may show FIGO grade 3 histology, intratumor heterogeneity, prominent lymphocytic infiltration, eosinophilic cytoplasmic changes, bizarre nuclei, and occasional serous-like or ambiguous features [54]. The ultramutated genotype generates abundant neoantigens and a strong antitumour immune response, providing a plausible basis for the AI-detected immune-rich morphology. However, these features are neither specific nor pathognomonic of this disease. In particular, the lymphocyte-rich phenotype substantially overlaps with MMRd/MSI tumours, indicating that AI may detect shared hypermutated phenotypes rather than POLEmut-specific morphological signatures [19,36].

#### 3.3.3. AI Performance of POLEmut Type

POLEmut tumours showed the greatest variability in performance across the models. Early one-versus-rest prediction with Panoptes showed only moderate discrimination for POLEmut, with an internal test AUROC of 0.681 [18]. The im4MEC subsequently achieved an AUROC of 0.849 in an independent PORTEC-3 cohort [19]. Hierarchical foundation model approaches, including hi-UNI, improved internal performance by combining tissue-scale architecture with cellular details, although independent external validation was not performed in this study [29]. Guo et al. reported an external AUROC of 0.798 in TCGA [36]. In larger real-world studies, external AUROCs ranged from 0.646 to 0.798, demonstrating that favourable internal performance (AUROC up to 0.849) does not consistently persist across independent clinical cohorts [34,35].

#### 3.3.4. Interpretability of POLEmut Type

Interpretability analyses have associated POLEmut predictions with solid growth, high-grade cytology, lymphocyte-rich, and spatially dispersed attention patterns, rather than being concentrated in high-activation regions [19,36]. Cell-level spatial analysis also suggests greater stromal and architectural heterogeneity in the TME of POLE tumours. These findings indicate that POLEmut prediction may depend on a combination of tumour cell atypia, immune contexture, and spatial heterogeneity rather than a single morphological hallmark. The diffuse and nonspecific nature of these signals partly explains the instability of POLEmut prediction performance.

#### 3.3.5. Major Limitations and Clinical Implications of POLEmut Type

POLEmut tumours remain an intrinsically challenging target for H&E-based molecular inference in EC. Its low prevalence and marked class imbalance limit the availability of training cases, and its morphological features may substantially overlap with those of MMRd/MSI tumours. Performance estimates are often based on a few positive cases; therefore, they have wide uncertainty. Intratumoural sampling variability further complicates predictions because models may rely on proxy signals, such as inflammation, grade, or solid growth, rather than POLE-driven molecular biology. Consequently, the sensitivity and positive predictive value may remain unstable, even when AUROC values are acceptable. AI may be used to prioritise cases for confirmatory POLE sequencing; however, it should not be used alone to support treatment de-escalation without conventional molecular assays.

### 3.4. NSMP

#### 3.4.1. Biological and Clinical Significance of NSMP Type

The NSMP subtype represents the largest category, accounting for approximately 30–40% of ECs [37]. Tumours are classified as NSMP when POLEmut, MMRd/MSI, and p53abn subtypes are excluded; therefore, this category encompasses biologically heterogeneous tumours without a single dominant molecular driver. Molecularly, NSMP tumours often harbour PI3K–AKT pathway alterations, show high oestrogen receptor (ER) or progesterone receptor (PR) protein expression, and contain CTNNB1 mutations, whereas subsets of this subtype lack detectable alterations using routinely assessed assays [4,5]. ER IHC is clinically relevant in this context, as ER status provides prognostic information within the NSMP subgroup and predicts response to endocrine therapy in advanced or recurrent disease [45]. From an AI perspective, the lack of a single defining molecular or morphological signature may make NSMP more difficult to infer from H&E-stained WSIs.

#### 3.4.2. Morpho-Molecular Correlates of NSMP Type

NSMP tumours commonly show well-formed endometrioid glands with smooth luminal borders and mild nuclear atypia, low lymphocytic density, focal squamous differentiation, and low tumour-to-stroma ratio [19]. Subsequent studies have reported preserved glandular morphology and increased stromal cellularity [19,28,36]. However, these features are nonspecific. Because NSMP is an exclusion-defined group, AI may learn residual low-grade morphology, stromal composition, or cohort-specific histotype distributions rather than a unique NSMP molecular phenotype.

#### 3.4.3. AI Performance of NSMP Type

NSMP prediction has shown moderate but cohort-dependent performance. The im4MEC model achieved an AUROC of 0.883 in the independent PORTEC-3 cohort [19]. Guo et al. [36] reported external AUROCs of 0.844 in TCGA and 0.873 in the Suzhou cohort. However, the performance declined to 0.684 and 0.770 in larger real-world cohorts [34,35]. Thus, although NSMP cases are relatively abundant, they may not produce stable performance because the reference category contains substantial biological and morphological heterogeneities.

#### 3.4.4. Interpretability of NSMP Type

NSMP tumours predicted as p53abn by AI showed increased nuclear atypia and worse outcomes, suggesting that some discordant predictions may reflect biologically meaningful p53abn-like morphology rather than random model error [19]. Darbandsari et al. [25] identified a p53abn-like subset of NSMP ECs associated with poorer survival than that of patients with residual NSMP tumours. This subgroup exhibited marked nuclear atypia and increased CNA despite a wild-type p53 IHC pattern, and some cases harboured TP53 mutations on sequencing. These findings suggest that H&E-based AI may identify adverse morphological and molecular programs within NSMP tumours that are not fully captured by conventional assays and provide clinical value by refining risk stratification in this heterogeneous tumour group.

#### 3.4.5. Major Limitations and Clinical Implications of NSMP Type

The NSMP group presents the potential limitations of morphology-based molecular inferences. Although many NSMP tumours exhibit apparently low-risk endometrioid morphology, this subgroup is biologically heterogeneous and encompasses a range of clinically relevant genomic alterations that may not be readily apparent on routine histopathological assessment. CTNNB1 mutations, which predominantly occur in NSMP tumours, activate Wnt/β-catenin signalling and may influence proliferation, differentiation, epithelial adhesion, polarity, and epithelial–mesenchymal transition. Clinically, CTNNB1-mutated NSMP tumours are important because they are associated with an increased risk of recurrence, despite otherwise low-risk histological features [55]. This discrepancy between favourable morphology and adverse molecularly driven behaviour suggests that the NSMP classification alone may be insufficient for an accurate risk assessment. Therefore, in NSMP tumours, AI may be more useful for risk refinement and identification of subtle differences in molecular-histological patterns than for simple NSMP subtype confirmation.

## 4. AI-Based Prediction of TMB in EC

### 4.1. Biological and Clinical Significance of TMB

TMB has been investigated as a biomarker for predicting the response to immune checkpoint inhibitor therapy in patients with cancer. As a quantitative measure of the somatic mutation load within a tumour, TMB reflects the potential capacity of cancer cells to generate neoantigens that can be recognised by the host immune system. Accordingly, highly mutated tumours may be more immunogenic and more susceptible to immune checkpoint blockade, particularly in molecular contexts such as MMRd/MSI or POLEmut EC [56].

In EC, TMB varies substantially across molecular subtypes: POLEmut tumours typically exhibit extremely high mutation burdens (>100 mutations/Mb), MMRd tumours generally show high TMB levels (10–100 mutations/Mb), whereas NSMP and p53abn tumours usually have low TMB (<10 mutations/Mb) [10]. However, despite its clinical relevance, TMB assessment requires comprehensive sequencing-based genomic profiling, which is costly, technically demanding, and not universally accessible in all laboratories. These limitations have motivated recent studies to explore whether DL models can directly infer TMB from routine H&E-stained WSIs.

### 4.2. AI Performance of TMB Prediction

Wang et al. [24] proposed a multilayer attention-based multiple-instance learning framework, termed TR-MAMIL, which used truncated ResNet feature encoders to analyse H&E-stained WSIs. The model was designed to classify ECs into histologically aggressive and non-aggressive groups and to predict TMB directly from routine histopathology image analysis. In the TCGA cohort, TR-MAMIL demonstrated a strong performance in distinguishing aggressive from non-aggressive histological groups, achieving an AUROC of 0.88. In contrast, TMB prediction showed substantial subgroup-dependent variability: the model achieved an AUROC of 0.82 for aggressive tumours but only 0.56 for non-aggressive tumours. These findings demonstrate clear task-dependent differences in the H&E-based TMB predictions.

### 4.3. Interpretability of TMB Prediction

The inference of aggressive versus non-aggressive histological types of EC was relatively robust, whereas TMB prediction was substantially more challenging, particularly in histologically non-aggressive tumours. This difference is biologically plausible because TMB is a sequencing-derived quantitative biomarker rather than a directly observable histological phenotype. Its relationship with morphology is likely indirect and mediated through associated features, such as tumour grade, immune infiltration, genomic instability, and molecular subtype composition. The subgroup-dependent performance of TR-MAMIL suggests that TMB-associated morphological signals may be more detectable in aggressive EC, whereas in non-aggressive tumours, such cues may be weaker, spatially dispersed, or less tightly coupled to visible morphology [24]. Therefore, poor performance in non-aggressive EC should not be interpreted solely as a modelling limitation but may also reflect inherent biological and morphological constraints on H&E-stained WSI-based TMB inference. Additionally, none of the models were validated in an independent non-TCGA cohort in the three studies [24,30,31]. Therefore, these studies should be interpreted as methodological comparisons within an overlapping public dataset, rather than as independent confirmations of clinical generalisability.

### 4.4. Major Limitations and Clinical Implications of TMB Prediction

The major limitations include confounding by molecular subtype, histotype, and grade; conversion of a continuous biomarker into a binary label; assay-dependent ground truth; repeated use of the same public cohort; and the predominance of frozen WSIs in the principal dataset, in which only 16% of the slides were FFPE slides. Calibration, biopsy validation, independent multicentre testing, and prospective evaluation against immunotherapy response are also lacking. Therefore, the most plausible near-term application is enrichment or prioritisation for confirmatory genomic TMB testing in patients with aggressive histological EC, particularly when sequencing capacity is limited.

## 5. Discussion

Overall, histology-based AI shows subtype-dependent rather than uniform utility. MMRd/MSI is the most extensively studied and relatively tractable target because it is routinely tested and often produces a lymphocyte-rich phenotype, although assay discordance and overlap with POLEmut remain important limitations [19,26]. POLEmut prediction is less stable because the incidence of POLE type in EC is low, and its high-grade and immune-rich morphology substantially overlaps with that of MMRd/MSI [19]. Dedicated evidence for p53abn tumours in EC is limited, but it is often the best-discriminated subtype. This suggests that marked nuclear atypia and serous-like architecture may provide more stable image signals than other molecular subtypes, although grade and histotype are confounding factors [19,25,34]. NSMP tumours are exclusion-defined and biologically heterogeneous; therefore, AI may be more valuable for intragroup risk refinement than for simple subtype confirmation [25]. TMB prediction remains challenging because TMB is a continuous sequencing-derived biomarker with indirect, subtype-confounded morphological correlates, and no study has shown independent external validation [24,30,31].

**Figure 3:**
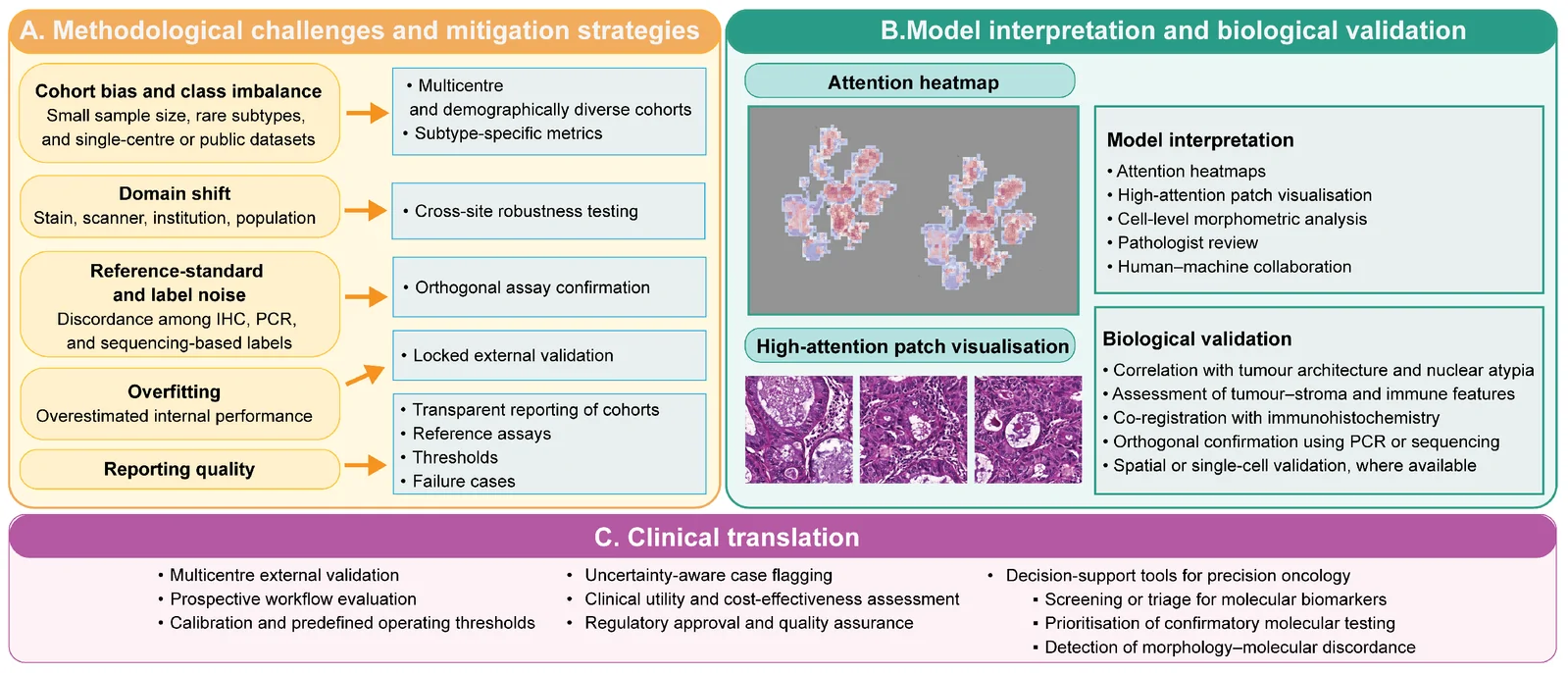
Methodological challenges, interpretability, and validation requirements for the clinical translation of WSI-based AI molecular classification in endometrial cancer. (**A**) Major methodological challenges include cohort bias and class imbalance, domain shift arising from institutional, staining, scanner, and population variability, reference standard and label noise, overfitting, and insufficient reporting transparency. These limitations can be addressed through multicentre and diverse cohorts, subtype-specific performance assessment, cross-institutional robustness testing, assay-specific and orthogonally confirmed reference standards, locked external test sets, and transparent reporting. (**B**) Attention heatmaps, high-attention patch visualisation, cell-level morphometric analysis, and pathologist review may facilitate model interpretation, whereas correlation with histomorphology features and orthogonal molecular or spatial assays can provide biological validation. (**C**) Clinical translation requires calibrated operating thresholds, prospective workflow evaluation, assessment of clinical utility and cost-effectiveness, regulatory approval, and quality assurance. The intended role of these models is to support biomarker screening, prioritisation of confirmatory molecular testing, and detection of morphology–molecular discordance, rather than replacing established molecular assays. *Abbreviations*: AI, artificial intelligence; IHC, immunohistochemistry; PCR, polymerase chain reaction; WSI, whole-slide image.

Several methodological limitations should be considered when interpreting AI-based molecular inferences for clinical translation (Figure 3).

### 5.1. Cross-Study Methodological Appraisal

The reported performance should not be interpreted as a direct ranking of the models because the included studies differed substantially in cohort composition, specimen type, analysis unit, reference standard, and validation design. Several studies used relatively small cohorts, including 95 patients in Zhang et al. [20], 114 eligible patients in Umemoto et al. [23], and 136 patients in Zhou et al. [32]. In addition, two studies relied mainly on a single-institution hold-out test or cross-validation without independent external validation [22,29]. Even when external validation was performed, the cohort size and composition varied; for example, Qi et al. [28] used two relatively small external cohorts of 35 and 83 patients. These differences affect statistical precision and may not contribute to estimating proper performance.

The reported sample units require careful interpretation. Patients, WSIs, individual slides, image tiles, and TMA cores are not equivalent. Multiple slides or thousands of tiles from one patient do not increase the number of biologically independent cases. Moreover, TMAs sample only small tumour regions and cannot fully capture the whole-tumour architecture or spatial heterogeneity. For example, Darbandsari et al. [25] used WSIs for model development, a TMA cohort for one external validation analysis, and multicentre WSIs for subsequent external validation; therefore, the performance across tissue types should be interpreted in the context of specimen sampling.

Validation design is another major determinant of reported performance. Cross-validation and held-out internal testing estimate performance within the source data distribution, whereas independent external validation assesses generalisability across institutions, scanners, staining workflows, and patient populations. Hong et al. [18] reported that several molecular prediction tasks showed lower performance in the independent NYU cohort than in internal testing. More recently, Wagner et al. [34] showed that conventional CNNs with internal macro-AUROCs of up to approximately 0.83 decreased to 0.53–0.59 in an external real-world cohort, whereas foundation model pipelines retained higher, albeit still moderate discrimination, with a maximum external macro-AUROC of 0.780. Schwarzmann et al. [35] further showed that additional stain normalisation did not consistently improve external performance, indicating that computational normalisation alone cannot eliminate domain shift.

Differences in preprocessing and learning strategies also contribute to the heterogeneity of performance. The studies varied in terms of tumour segmentation, inclusion of non-tumour tissue, patch size, magnification, colour normalisation, image quality filtering, and aggregation of patch-level predictions. Some approaches assign case-level labels to all selected patches, whereas weakly supervised multiple-instance learning uses only slide- or bag-level labels and allows attention or pooling mechanisms to identify informative regions. Multi-resolution CNNs, attention-based MIL, transformer aggregators, and foundation model feature extractors solve related but methodologically distinct tasks.

Ground truth heterogeneity is equally important. MMR-related labels were derived from full or reduced IHC panels, PCR-based MSI testing, NGS-based MSI assessment, or TCGA multi-omics classification. Similarly, p53abn defined by IHC is closely related to, but not identical to, sequencing-defined TP53 mutations. Discordance may result from technical variation, interpretative differences, subclonal alterations, heterogeneous protein expression, multiple-classifier tumours, or testing of an imaged slide from a different block. Future studies should report the exact reference method, analyse assay-defined groups separately where feasible, and use the same tissue block or spatially matched regions for molecular testing and image analysis.

The AUROC measures discrimination across possible thresholds but does not define a clinically acceptable operating point. For example, MMRNet achieved an AUROC of 0.897 but a sensitivity of only 0.628 at the reported threshold, illustrating that high discrimination does not necessarily ensure safe screening or prioritisation performance [26]. Therefore, AUROC should not be used in isolation to rank studies or determine clinical readiness; threshold-dependent performance, calibration, and independent external evaluation should be considered together, as detailed in Section 5.3 and Table 3.

### 5.2. Interpretability and Biological Validation

Interpretability is essential for assessing the biological validity and clinical credibility of AI-based molecular inferences (Figure 3B) [57,58]. Attention heatmaps and other visualisation methods can provide partial insights into the model behaviour by highlighting the image regions that contribute to the prediction. However, such visual explanations do not automatically establish the biological plausibility of the models. Model-highlighted regions should be systematically reviewed by expert pathologists to determine whether they correspond to diagnostically or biologically meaningful features, such as tumour morphology, immune infiltration, stromal remodelling, and necrosis, rather than irrelevant artefacts, including tissue folds, staining variation, out-of-focus areas, pen marks, debris, or slide preparation effects [59].

More rigorous validation should combine heatmaps with quantitative cellular and spatial analyses. Guo et al. [36] extended this approach using nuclear segmentation and single-cell morphometric and spatial features. Darbandsari et al. [25] further showed that an AI-defined p53abn-like NSMP subgroup had increased CN abnormalities and adverse clinical outcomes, providing orthogonal genomic and prognostic validations. Therefore, future studies should perform genomic, transcriptomic, or immunophenotypic validation in the same block or model-highlighted region to distinguish true morphology–molecular associations from cohort-level confounding factors.

### 5.3. Clinical Translation and Deployment

The clinical role of H&E-based AI for molecular prediction in EC should be defined by the intended use, molecular target, and clinical consequences of prediction errors, rather than by the overall AUROC alone. A practical pathology workflow would comprise slide quality and tumour adequacy assessment, followed by the generation of subtype probabilities, heatmaps, and an abstention option, pathologist review, subtype-specific confirmatory testing, and final assay-based molecular classification. Confirmatory laboratory results should remain the basis for diagnostic and treatment decisions.

The preferred operating objectives and thresholds differ among the molecular targets (Table 4). For MMRd/MSI, AI is most plausibly used as a high-sensitivity, high-NPV prioritisation tool to identify cases requiring prompt IHC, PCR, or NGS. Therefore, the threshold could be set at the highest value that meets the predefined sensitivity and NPV criteria at the intended-use prevalence, while reporting the number of missed cases per 1000 patients and the confirmatory testing referral rate. For p53abn, a high specificity and high PPV rule-in alert could prompt p53 IHC or TP53 testing in morphologically ambiguous tumours; the operating point could be selected to meet predefined specificity and PPV criteria while limiting false alerts. For POLEmut, a high-sensitivity referral threshold may prioritise sequencing and minimise missed cases, with both missed-case and sequencing referral rates reported. For NSMP tumours, AI may be more useful for risk refinement and the discovery of high-risk morpho-molecular patterns than for simple subtype confirmation; therefore, calibration across prespecified risk strata and clinical net benefit assessed using decision curve analysis are more informative than a single binary cutoff. AI-predicted TMB should be restricted to enrichment for genomic testing and should not independently determine immunotherapy eligibility. Human–AI fusion in MMRNet and the identification of p53abn-like NSMP tumours support this subtype-specific decision-support framework [25,26].

Because universally accepted numerical cutoffs are unavailable, operating rules should be specified before independent test data are examined rather than selected post hoc to maximise overall accuracy. Study protocols should define the target population and specimen type, reference standard, expected prevalence in the intended-use population, clinical action triggered by the prediction, relative consequences of false-negative and false-positive results, numerical acceptance criteria, and threshold-selection rule.

Probability calibration and threshold selection should use development data only, either a prespecified calibration subset or the inner loop of an appropriately nested resampling procedure. When cross-validation is used, all model development steps, including preprocessing, model fitting, probability calibration, and threshold selection, should be repeated within the training portion of each fold. The model version, preprocessing pipeline, calibration mapping, operating threshold, and abstention rule should then be locked before internal testing and independent external evaluation. External cohorts should have no patient overlap with the development data, originate from distinct institutions and/or time periods, and represent the intended clinical setting. The primary external analysis should apply the locked pipeline without threshold re-optimisation and report calibration and threshold-dependent performance separately for each cohort. If local recalibration is performed, pre- and post-recalibration performance should be presented separately, and the updated model should undergo further independent evaluation. The minimum reporting and evaluation requirements are summarised in Table 3, in accordance with the reporting principles of TRIPOD + AI and STARD-AI and the methodological safeguards of PROBAST + AI [60,61,62].

Analytical validation should define acceptable fixation and staining conditions, scanner compatibility, minimum tumour area, image quality failure criteria, and performance across biopsy, curettage, and hysterectomy specimens. The software should record the model version and AI results used for each case, allow pathologists to correct or reject AI predictions, and undergo regular quality checks to ensure stable performance after its deployment. Regulatory approval, laboratory accreditation, quality assurance, data governance, reimbursement, and cost-effectiveness must be considered before routine implementation.

Clinical translation should proceed sequentially through retrospective multicentre validation, human–AI workflow studies, clinical impact trials and controlled routine deployment. Prospective evaluations should extend beyond model-level performance metrics to assess whether AI-assisted workflows improve diagnostic efficiency, prioritisation and allocation of confirmatory testing without increasing missed cases, shorten turnaround times, support treatment selection, and maintain patient safety in the clinical setting. Overall, H&E-based AI is best positioned as a calibrated triage and decision-support tool that complements, rather than replaces, guideline-recommended molecular testing.

### 5.4. Future Research Roadmap

Near-term priorities should focus on establishing model generalisability, clinical safety, and workflow utility. These priorities include large multicentre external validation studies, prespecified operating thresholds, and rigorous assessment of calibration. Treatment planning for EC frequently begins with a fragmented preoperative curettage specimen; however, most current models have been developed using hysterectomy specimens. Umemoto et al. [23] noted that curettage-based models could support earlier treatment planning but would require separate development and validation because curettage specimens frequently show fragmentation and deformation. Therefore, a paired evaluation of curettage and hysterectomy specimens from the same patient is required. Future studies should consider multi-block sampling and spatially matched molecular reference standards to assess intratumor heterogeneity and sampling effects.

**Figure 4:**
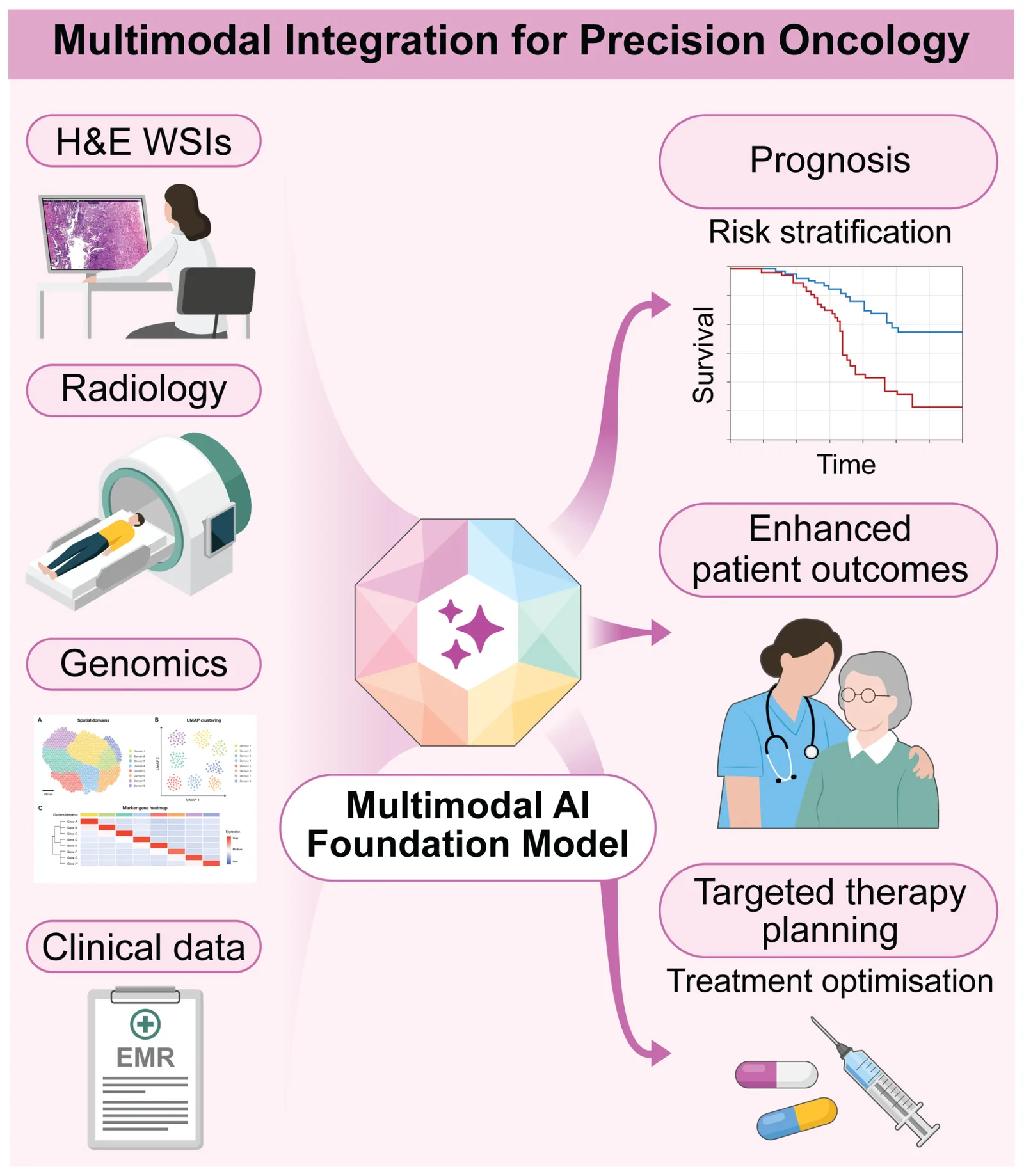
Multimodal integration of AI for precision oncology in endometrial cancer. Multimodal integration of AI and computational pathology for clinical translation. H&E-stained WSIs, radiomic and genomic data, and clinical information can be integrated within foundation model-based frameworks to support personalised prognosis, risk stratification, treatment optimisation, targeted therapy planning, and improved patient outcomes. Abbreviations: AI, artificial intelligence; H&E, haematoxylin and eosin; WSI, whole-slide image.

Future research should move beyond single-task molecular subtype classification toward clinically integrated decision-support systems. Medium- and long-term studies should evaluate EC-specific foundation models and multimodal systems. Models combining H&E histology with clinical variables, molecular data, radiological features, treatment and outcome data, and natural language pathology reports may better reflect real-world decision-making in EC management (Figure 4). Multimodal integration may further enhance risk prediction in biologically heterogeneous tumours. HECTOR, developed by Volinsky-Fremond et al. [17], integrates H&E-based WSI-derived histology, im4MEC-derived molecular class predictions, and anatomical stage to predict postoperative distant recurrence risk and identify patients who may benefit from adjuvant therapy in EC. Its superior performance over H&E-only DL models suggests that combining image-derived molecular information and clinical staging provides additive prognostic value. Such approaches could support not only molecular subtype prediction but also recurrence risk assessment, treatment stratification, and prediction of therapeutic response [17,63].

Foundation models pretrained on large-scale histopathology datasets represent another important research direction. By learning generalisable tissue representations, these models may improve data efficiency, reduce the need for task-specific training from scratch, and support diverse downstream applications, including molecular subtype prediction, biomarker discovery, recurrence risk prediction, and treatment response modelling [59,63,64]. Foundation model pipelines have shown better external robustness than conventional CNNs; however, their real-world performance remains moderate and does not eliminate the need for task-specific calibration, external validation, and prospective clinical evaluation [34,35].

Emerging single-cell, spatial transcriptomics, and multi-omic technologies may further enhance the biological interpretability of AI-based molecular inferences. Spatial transcriptomics, single-cell profiling, and multiplex immunofluorescence can help link image-derived features with the underlying molecular programs, immune contexture, and tumour–stroma interactions [65,66]. Explainable machine-learning frameworks that integrate quantitative histomorphology with molecular profiles may further support the identification and validation of clinically meaningful morpho-molecular associations [67]. These approaches may be particularly valuable for biologically heterogeneous molecular categories and morphologically overlapping tumours, such as immune-rich MMRd/MSI and POLEmut tumours.

### 5.5. Limitations of This Review

In this study, quantitative synthesis was not performed because the included studies differed substantially in terms of prediction targets, patient populations, specimen types, ground truth assays, data units, validation designs, and performance metrics. In addition, although the structured search and predefined eligibility criteria reduced potential selection bias, residual publication bias inherent in the published literature cannot be completely excluded. Because the AI literature is evolving rapidly, relevant studies published after the final search date may not be represented in this review.

## 6. Conclusions

The application of AI to digital pathology in EC has the potential to complement conventional assays for molecular classification and broaden access to molecularly informed care. Its greatest near-term value is likely to lie in calibrated triage, risk stratification, and the discovery of clinically meaningful image-based biomarkers rather than replacing established molecular assays. Safe clinical translation requires robust external validation and prospective evaluation of the impact of AI-assisted workflows. Ultimately, AI-driven digital pathology may serve as a bridge between routine pathology workflows and precision oncology, supporting more accessible, patient-centred, and molecularly informed treatment of EC.

## Figures and Tables

**Table 1:** Overview of research on AI-based molecular classification using WSIs of endometrial cancer.

Author (Year)	Model/Architecture	Internal Cohort/Dataset	External Cohort/Dataset	Task	Internal Performance	External Performance
Hong et al. (2021) [18]	“Panoptes”, custom multi-resolution, InceptionResNet-based CNN (2.5×, 5×, 10×)	TCGA and CPTAC, 496 WSIs of 456 pts, (train/val/test 8:1:1)	NYU, 137 WSIs of 41 pts	MSI	AUC 0.827	AUC 0.667
				CNV-H, CNV-L, POLE, TP53 (+17 genes) (GT: multi-omics, including sequencing)	CNV-H/TP53/POLE/CNV-L, AUC 0.934/0.873/0.681/0.889, POLE (multi-model), 0.89	CNV-H/TP53/POLE/CNV-L, AUC 0.795/0.920/NA/0.850
Fremond et al. (2023) [19]	“im4MEC”, SSL-MoCo-v2 + ResNet50, attention, HoVer-Net	PORTEC and multiple clinical cohorts, total 2028 pts, 4-fold CV	PORTEC-3, 393 pts	MSI	N/A	AUC 0.844
				4-class ProMisE (GT: molecular data from previous studies)	Macro-average AUC 0.874	Macro-average AUC 0.876, TP53/POLE/NSMP, AUC 0.928/0.849/0.883
Zhang et al. (2023) [20]	ResNet34, GAM-VGG16	TCGA, 95 WSIs of 95 pts, (train/test 70:25)	N/A	MSI (GT: multi-omics, including sequencing)	AUC/Acc/Sens/F1-score, 0.799/0.80/0.857/0.826	N/A
Wang et al. [npjDM] ^a^ (2024) [21]	Weakly supervised DL with FPS + MFCN + IPS + InceptionV3 + WSID	TCGA, 529 pts, (train/test ⅔:⅓)	N/A	MSI (GT: NGS)	Acc/Prec/Sens/F-measure, G1G2, 0.94/0.93/1.00/0.96; G3, 0.84/0.81/0.94/0.87, Inference time (1.03 s/WSI)	N/A
Whangbo et al. (2024) [22]	Multi-resolution ensemble, ImageNet, EfficientNetB2 (2.5×, 5×, 10×)	GUGMC, 1168 WSIs of 325 pts, (train/test 8:2)	N/A	MMRd (GT: 4 IHC)	AUC/Acc/Sens/Spec, 0.821/0.778/0.827/0.764	N/A
Umemoto et al. (2024) [23]	ResNet50, API-Net-based	SMUH, 114 pts, (train/val/test 70:15:15)	N/A	MMRd (GT: PMS2, MSH6 IHC)	AUC/Acc/Prec/Recall/F-score (per-tile level), ResNet50: 0.91/0.79/0.89/0.65/0.75; API: 0.85/0.85/0.75/0.69/0.72	N/A
Wang et al. [npjPO] ^a^ (2024) [24]	Truncated ResNet50 (for subtype), Truncated ResNet152 (for TMB), ImageNet-pretrained TR-MAMIL	TCGA, 918 WSIs of 529 pts for TMB; TSGH, TMA, 242 cores for MMRd and p53abn, [train/val/test (⅔ × 0.9: ⅔ × 0.1): ⅓]	N/A	MMRd(GT: IHC)	MLH1/MSH2/MSH6/PMS2, MeanSS: 0.92/0.83/0.83/0.84	N/A
				TMB-high/low by 10 mut/Mb (GT: NGS), p53abn (GT: IHC)	AUC/MeanSS, TMB in aggressive type 0.82/0.73; TMB in non-aggressive 0.56/0.68, p53 (TMA) 0.78/0.78, TP53 0.68/0.73	N/A
Darbandsari et al. (2024) [25]	VarMIL, ResNet34	TCGA, 155 WSIs of 146 pts; TU, 431 WSIs of 222 pts, 10-fold CV, (train/val/test per fold 60:20:20)	BC, TMA, 290 pts; CC 640 WSIs of 614 pts from 26 hospitals	NSMP, p53abn, p53abn-like NSMP (GT: IHC for p53abn)	p53abn vs. NSMP, AUC/Acc, 0.95/0.894	p53abn vs. NSMP, AUC/Acc, BC 0.88/0.798; CC 0.88–0.95/0.663–0.885
Liu et al. (2025) [26]	“MMRNet”, ensemble, EfficientNet, ResNet18, reader study, human–machine fusion	Internal-UCEC, 1027 WSIs of 1026 pts, 5-fold CV	TCGA, 401 WSIs of 369 pts; MultiCenter, 230 WSIs of 230 pts; GWCH, 421 WSIs of 421 pts	MMRd (GT: IHC)	AUC/Sens/Spec/NPV, 0.897/0.628/0.949/0.892	MMRNet (3 cohorts) AUC 0.790;0.807;0.863; H-M fusion (3 cohorts) AUC 0.802;0.913;0.932
Wang et al. [CMIG] ^a^ (2025) [27]	“IMAN”, SwAV-SSL-ResNet50	TSGH, TMA, 242 cores, (train/test ⅔:⅓)	N/A	MMRd biomarkers (GT: IHC)	MLH1/MSH2/MSH6/PMS2, MeanSS 0.85/0.92/0.91/0.92, Inference time (18.71 s/WSI)	N/A
				p53abn (GT: IHC)	MeanSS 0.81	N/A
Qi et al. (2025) [28]	SRResGAN, MedSAM, ResNet101, Grad-CAM	393 pts, (train/test 8:2)	OGHFU, 83 pts; PMCHH, 35 pts	MMRd (GT: IHC)	AUC 0.98	AUC, O: 0.96/P: 0.93
				4-class ProMisE (GT:sequencing for POLE, IHC for p53abn)	average AUC/Sens/Spec/overall Acc, 0.98/0.91/0.92/0.91, p53abn/POLE/NSMP, AUC 0.98/0.99/0.98	AUC/Sens/Spec/overall Acc O: 0.97/0.87/0.94/0.92 P: 0.94/0.94/0.90/0.93 p53abn/POLE/NSMP, AUC O: 0.97/0.98/0.94; P: 0.94/0.97/0.91
Cui et al. (2025) [29]	DeepLab v3, hi-UNI foundation model	FUSCC, 364 WSIs of 324 pts, 5-fold CV	N/A	MSI (GT: NGS)	Macro-average AUC 0.829	N/A
				4-class ProMisE (GT: NGS)	Macro-average AUC 0.879, TP53/POLE/NSMP, AUC 0.899/0.886/0.899	N/A
Wang et al. [MIA] ^a^ (2025) [30]	ETMIL-SSLViT	TCGA 918 WSIs of 529 pts, (train/test ⅔:⅓)	N/A	histologic subtype, TMB (GT: from TCGA dataset)	AUC/MeanSS, TMB in aggressive type 0.82/0.77; TMB in non-aggressive 0.61/0.64, Inference time (26.8 s/WSI)	N/A
Wang et al. [BSPC] ^a^ (2025) [31]	InceptionV3	TCGA 529 pts; TSGH, TMA, 242 cores for MMR biomarkers, (train/test ⅔:⅓)	N/A	MMRd biomarkers (GT: IHC)	MLH1/MSH2/MSH6/PMS2, AUC 0.947/0.889/0.905/0.894, MeanSS 0.89/0.84/0.81/0.81	N/A
				TMB-high/low, by 10 mut/Mb (GT: NGS)	AUC/MeanSS, TMB in aggressive type 0.734/0.76; TMB in non-aggressive 0.555/0.61, Inference time (0.37 s/WSI)	N/A
Zhou et al. (2025) [32]	“DLPRM” (multimodal), (ResNet50 + Pyradiomics)	136 pts (train/val 7:3)	N/A	MMRd (GT: 4 IHC)	Train/val AUC 0.960/0.917	N/A
Lee et al. (2025) [33]	ResNet50 + EfficientNet-b0; ResNet18; VGG19; ConvNext; NAT	TCGA 430 pts, 5-fold CV (train/test 8:2)	CPTAC 74 pts	MSI (GT: PCR)	AUC 0.69, Multi tissue trained model, AUC 0.79	AUC 0.76; Multi tissue trained model, AUC 0.77
Wagner et al. (2026) [34]	Virchow2 with CLAM MIL, UNI2 with CLAM MIL, (foundation model)	TCGA and CPTAC, 1195 WSIs of 815 pts 5-fold CV	1357 WSIs of 720 pts	MMRd (GT: IHC)	AUC 0.792	AUC 0.759
				4-class ProMisE (NGS for TP53, POLE)	Macro-AUC/macro-F1/balanced Acc, 0.860/0.607/0.647, p53abn/POLE/NSMP, AUC 0.942/0.849/0.869	Macro-AUC/macro-F1/balanced Acc, 0.780/0.416/0.507, p53abn/POLE/NSMP, AUC 0.851/0.798/0.770
Schwarzmann et al. (2026) [35]	CTransPath or UNI foundation model with STAMP protocol, Grad-CAM	TCGA, 529 pts; CPTAC, 254 pts 10-fold CV (train/val/test 64:16:20)	Erlangen, 332 pts	MMRd (GT: IHC)	Mean AUC, 0.719	Mean AUC, 0.700
				4-class ProMisE (GT: IHC for p53, sequencing for POLE)	p53abn/POLE/NSMP, Mean AUC 0.899/0.743/0.842	p53abn/POLE/NSMP, Mean AUC 0.844/0.646/0.684
Guo et al. (2026) [36]	DeepLab-v3, EfficientNetV2, Grad-CAM, human-in-the-loop triage tool	Fudan 364 WSIs of 324 pts, 5-fold CV	TCGA, 296 WSIs of 274 pts; Suzhou, 36 WSIs of 33 pts	MSI (GT: NGS, IHC)	AUC 0.846	AUC, T: 0.775; S: 0.761
				4-class ProMisE (GT: NGS, IHC)	Macro-average AUC 0.867, TP53/POLE/NSMP, AUC 0.910/0.835/0.876	Macro-average AUC, T: 0.844; S: 0.847, TP53/POLE/NSMP, AUC, T: 0.950/0.798/0.844; S: 0.862/NA/0.873

^a^, all marked studies were first authored by Wang et al.; journal abbreviations are provided to distinguish these studies. BSPC, Biomedical Signal Processing and Control; CMIG, Computerized Medical Imaging and Graphics; MIA, Medical Image Analysis; npjDM, npj Digital Medicine; npjPO, npj Precision Oncology. Abbreviations: Acc, accuracy; AI, artificial intelligence; AUC (in this table, AUC is used synonymously with AUROC), area under the receiver operating characteristic curve; CNN, convolutional neural network; CNV-H, copy number high; CNV-L, copy number low; CV, cross-validation; DL, deep learning; GT, ground truth; H-M fusion, human–machine fusion; IHC, immunohistochemistry; MeanSS, mean sensitivity and specificity; MIL, multiple instance learning; MMR, mismatch repair; MMRd, mismatch repair deficiency; MSI, microsatellite instability; NGS, next-generation sequencing; NPV, negative predictive value; NSMP, no specific molecular profile; p53abn, aberrant p53 expression; POLE, pathogenic POLE exonuclease domain mutations; Prec, precision; ProMisE, Proactive Molecular Risk Classifier for Endometrial Cancer; pts, patients; Sens, sensitivity; Spec, specificity; test, test dataset; TMB, tumour mutational burden; train, training dataset; val, validation dataset; WSI, whole slide image. Model- and method-related abbreviations: DLPRM, deep learning pathoradiomics model; ETMIL-SSLViT, ensemble transformer-based multiple instance learning with self-supervised learning vision transformer feature encoder; FPS, foreground patch selection; GAM, GRU-based attention module; GRU, gated recurrent unit; hi-UNI, hierarchical UNI; im4MEC, image-based four molecular classes in endometrial cancer; IMAN, weakly supervised interpretable multi-stage attention deep learning network; IPS, iterative patch sampling; MFCN, modified fully convolutional network; SSL, self-supervised learning; SwAV, swapping assignments between multiple views; TR-MAMIL, truncated ResNet-based multilayer attention multiple instance deep learning framework; WSID, weighted softmax integrated decision. Cohort abbreviations: BC, British Columbia cohort; CC, Cross Canada cohort; CPTAC, Clinical Proteomic Tumor Analysis Consortium cohort; FUSCC, Fudan University Shanghai Cancer Center cohort; GUGMC, Gachon University Gil Medical Center cohort; GWCH, Guangdong Women and Children Hospital cohort; NYU, New York University cohort; OGHFU, Obstetrics and Gynecology Hospital of Fudan University cohort; PMCHH, Pingdingshan Maternal and Child Health Hospital cohort; SMUH, Sapporo Medical University Hospital cohort; TCGA, The Cancer Genome Atlas; TSGH, Tri-Service General Hospital cohort; TU, Tübingen University cohort; UCEC, uterine corpus endometrial carcinoma cohort.

**Table 2:** Common computational pathology terms.

Term	Definition
CNN	A neural network that uses learned convolutional filters to extract image features.
SSL	Self-supervised learning: Representation learning from unlabelled images using pretext or contrastive objectives without task-specific manual labels.
MIL	Multiple-instance learning: Slide-level labels supervise a bag of image patches without requiring a label for every patch.
Attention-based MIL	MIL assigns relative weights to individual patches when generating slide-level predictions.
Transformer	An architecture that uses attention mechanisms to determine the relationships among image tokens or patches.
Foundation model	A large pretrained encoder that is adapted or fine-tuned for downstream pathology tasks.
Multimodal model	A model that integrates histology with clinical, molecular, radiological, or textual information.

**Table 3:** Key performance metrics and minimum reporting requirements.

**A. Interpretation of common performance metrics.**	
Metric	Interpretation
AUROC	Threshold-independent ranking discrimination; it does not define a clinically usable operating point.
Sensitivity	Proportion of positive cases detected; central to rule-out or case-prioritisation safety.
Specificity	Proportion of negative cases correctly excluded; central to rule-in alerts and referral burden.
PPV/NPV	The probability that a positive/negative prediction is correct; both depend on target prevalence.
F1-score	Harmonic mean of precision and recall; useful with class imbalance but insensitive to true negatives.
Balanced accuracy/meanSS	Average performance across classes or between sensitivity and specificity; useful for imbalanced data.
C-index	Ability of a prognostic model to rank time-to-event risk; not a classification probability.
Calibration	Agreement between predicted probabilities and observed outcome frequencies.
Net benefit	Clinical utility after weighting true-positive and false-positive decisions at a selected threshold.
**B. Minimum reporting requirements for operating thresholds.**	
Domain	Minimum requirement
Intended use	Target population and specimen, reference standard, expected prevalence, clinical action, and relative importance of false-negative and false-positive results.
Threshold selection	Exact threshold, prespecified selection rule, development dataset used, and any abstention or indeterminate range.
Performance and calibration	Observed prevalence, confusion matrix, sensitivity, specificity, PPV, NPV, and 95% CIs; calibration plot; intercept; slope; and Brier score.
Clinical burden	Test-positive or referral rate, false-positive and false-negative cases per 1000 patients, and technical failure or abstention rate.
Independent external evaluation	Apply the locked model, preprocessing, calibration, and threshold without re-optimisation to patient-independent data from a distinct institution and/or time period; report site-specific and relevant subgroup performance.
Updating and utility	Report performance before and after recalibration separately, together with decision curve net benefit and tests triggered, avoided, or missed.

The AUROC alone is insufficient for clinical interpretation; calibration, threshold-dependent performance, prevalence, and independent external evaluation should be considered together.

**Table 4:** Molecular subtype-specific predictability, evidence maturity, and clinical translational potential of H&E-based AI in endometrial cancer.

Molecular Subtype	Biological Visibility	Data Availability/Evidence Maturity	Model Performance	Principal Challenge	Potential Clinical Application/Clinical Maturity
MMRd/MSI	Moderate–high: lymphocyte-rich, inflamed morphology	High: most extensively studied, with multicentre validation	Moderate, but variable across cohorts	Assay discordance; overlap high-inflamed morphology with POLEmut; domain shift	Triage for confirmatory MMR IHC or MSI testing; highest relative maturity
p53abn	High: marked nuclear atypia and serous-like architecture	Moderate: repeatedly assessed, but fewer dedicated studies	Generally highest and most stable	Assay discordance; grade and histotype confounding	Rule-in flag for p53 IHC or TP53 testing; moderate maturity
POLEmut	Low–moderate: immune-rich or high-grade features are nonspecific	Low: rare subtype with limited external validation	Variable; often weakest	Low prevalence, class imbalance, and overlap morphologic features with MMRd/MSI	Prioritisation for POLE sequencing; low maturity
NSMP	Moderate: low-grade endometrioid tumor but nonspecific features	Moderate: common but heterogeneous, exclusion-defined subtype	Moderate and cohort-dependent	Biological and label heterogeneity	Intragroup risk refinement rather than subtype confirmation; emerging maturity

All proposed clinical applications require intended-use-specific operating criteria that are prespecified, calibrated, and independently validated; the minimum reporting requirements are summarised in Table 3.

## Data Availability

No new data were created or analyzed in this study. Data sharing is not applicable to this article.

## Supplementary Materials

The following supporting information can be downloaded at: https://www.mdpi.com/article/10.3390/ijms27167341/s1.

## Abbreviations

AI	Artificial intelligence
AUROC	Area under the receiver operating characteristic curve
CN	Copy number
CNA	Copy number alteration
DL	Deep learning
EC	Endometrial cancer
ER	Oestrogen receptor
H&E	Haematoxylin and eosin
IHC	Immunohistochemistry
MMR	Mismatch repair
MMRd	Mismatch repair deficiency
MSI	Microsatellite instability
NGS	Next-generation sequencing
NSMP	No specific molecular profile
p53abn	Aberrant p53 expression
POLEmut	Pathogenic POLE exonuclease domain mutation
PR	Progesterone receptor
ProMisE	Proactive Molecular Risk Classifier for Endometrial Cancer
TCGA	The Cancer Genome Atlas
TMB	Tumour mutational burden
TME	Tumour microenvironment
WSIs	Whole-slide images

## Appendix A

**Table A1:** Major strengths and Limitations of the Studies Included in Table 1.

Author (Year)	Major Strength and Principal Limitation
Hong et al. (2021) [18]	Strength: Early multi-resolution, multi-target WSI framework with an independent NYU cohort. Limitation: One-versus-rest tasks and small external cohort.
Fremond et al. (2023) [19]	Strength: Large trial-linked cohort, interpretable attention MIL, and independent PORTEC-3 validation. Limitation: POLEmut was frequently confused with image-based MMRd.
Zhang et al. (2023) [20]	Strength: A stepwise, interpretable MSI prediction pipeline integrating tumour-region selection. Limitation: Only 95 TCGA cases and no external validation.
Wang et al. [npjDM] ^a^ (2024) [21]	Strengths: Fast inference and grade-stratified MSI evaluation. Limitation: TCGA-only internal split; overlapping TCGA evidence with no external validation.
Whangbo et al. (2024) [22]	Strength: Aligned multi-resolution tumour-region ensemble in a relatively large WSI set. Limitation: Single institution, pathologist ROI dependence, and no external validation.
Umemoto et al. (2024) [23]	Strength: Systematic comparison of multiple CNN and attention-based models with patient-level splitting. Limitation: Small single-centre, endometrioid-only cohort with tile-level evaluation and no external validation.
Wang et al. [npjPO] ^a^ (2024) [24]	Strength: Integrated TMA biomarker and WSI TMB tasks with attention MIL. Limitation: No external validation; TMA sampling and reuse of TCGA/TSGH cohorts limit independence.
Darbandsari et al. (2024) [25]	Strength: Multicentre validation and discovery of a reproducible p53abn-like NSMP phenotype. Limitation: Specimen-format differences (WSI versus TMA).
Liu et al. (2025) [26]	Strength: Three external cohorts, reader studies, and human–machine fusion. Limitation: Assay/site heterogeneity and reduced standalone sensitivity at the reported operating point.
Wang et al. [CMIG] ^a^ (2025) [27]	Strength: Hierarchical attention at the slide, patch, and cell levels. Limitation: Single TSGH TMA cohort, no external validation, and limited whole-tumour architecture.
Qi et al. (2025) [28]	Strength: Two external institutional cohorts, lesion segmentation, and four-class evaluation. Limitation: Small external cohorts and limited evidence that thresholds generalise without recalibration.
Cui et al. (2025) [29]	Strength: EC-specific hierarchical foundation model with high internal four-class discrimination. Limitation: Single-centre fivefold cross-validation and no independent external cohort.
Wang et al. [MIA] ^a^ (2025) [30]	Strength: Transformer MIL and task-stratified TMB analyses. Limitation: Reused TCGA cohort, internal-only validation, and weak performance in non-aggressive tumours.
Wang et al. [BSPC] ^a^ (2025) [31]	Strength: Joint assessment of MMR proteins and TMB with rapid inference. Limitation: Overlapping TCGA/TSGH data, TMA sampling, and no external validation.
Zhou et al. (2025) [32]	Strength: Multimodal deep learning/radiomics model. Limitation: 136 patients; internal training–validation split only.
Lee et al. (2025) [33]	Strength: Independent CPTAC testing and multi-cancer transfer analysis. Limitation: Moderate EC-specific AUROC.
Wagner et al. (2026) [34]	Strength: Direct comparison of encoders and MIL aggregators with large external validation. Limitation: External macro-F1 and balanced accuracy remained modest despite the improved AUROC.
Schwarzmann et al. (2026) [35]	Strength: Consecutive real-world cohort, scanner/stain analyses, and foundation model comparison. Limitation: Moderate four-class discrimination; stain normalisation did not resolve the domain shift.
Guo et al. (2026) [36]	Strength: Two independent cohorts and a human-in-the-loop triage design. Limitation: Small Suzhou cohort and absence of POLEmut positives.

^a^, all marked studies were first authored by Wang et al.; journal abbreviations are provided to distinguish these studies. BSPC, Biomedical Signal Processing and Control; CMIG, Computerized Medical Imaging and Graphics; MIA, Medical Image Analysis; npjDM, npj Digital Medicine; npjPO, npj Precision Oncology.
