# Supplementary material for: Artificial Intelligence and Digital Pathology for Molecular Classification of Endometrial Cancer
Source: Int J Mol Sci. 2026 Aug 17;27(16):7341. doi: 10.3390/ijms27167341 (PMC13512974; doi:10.3390/ijms27167341)
Supplement: Supplementary file 1 [file ijms-27-07341-s001.zip › ijms-4437159-supplementary.pdf]

International Journal of Molecular Sciences

Supplemental information

Artificial Intelligence and Digital Pathology for Molecular Classification of Endometrial Cancer

Yesul Jeong [1], Sungman Hong [2], Sangjeong Ahn [2,3,*] and Sung Hak Lee [4,*]

1 Department of Hospital Pathology, St. Vincent's Hospital, College of Medicine,

The Catholic University of Korea, Seoul, 06591, Republic of Korea; seulyool@catholic.ac.kr

2 Department of Biomedical Informatics, College of Medicine, Korea University, Seoul, 02841,

Republic of Korea; sungmanhong@gmail.com

3 Department of Pathology, College of Medicine, Korea University, Seoul, 02841, Republic of Korea

4 Department of Hospital Pathology, Seoul St. Mary's Hospital, College of Medicine,

The Catholic University of Korea, Seoul, 06591, Republic of Korea

* Correspondence: vanitas80@korea.ac.kr (S.A.); hakjjang@catholic.ac.kr (S.H.L.); Tel.: +82-10-3571-9887 (S.A.); +82-10-7743-8580 (S.H.L.)

Table S1. Database-specific search strategies

A. **PubMed**

("Endometrial Neoplasms"[Mesh] OR "Endometrial Neoplasms"[TW] OR "Endometrial Neoplasm"[TW] OR "Endometrial Carcinoma"[TW] OR "Endometrial Carcinomas"[TW] OR "Endometrial Cancer"[TW] OR "Endometrial Cancers"[TW]) AND ("Artificial Intelligence"[Mesh] OR "Artificial Intelligence"[TW] OR "AI"[TW] OR "Machine Learning"[Mesh] OR "Machine Learning"[TW] OR "Transfer Learning"[TW] OR "Deep Learning"[Mesh] OR "Deep Learning"[TW] OR "Hierarchical Learning"[TW] OR "Ensemble Learning"[TW] OR "Multimodal Deep Learning"[TW] OR "Foundation Model"[TW] OR "Foundation Models"[TW]) AND ("Molecular Classification"[TW] OR "Molecular Classifications"[TW] OR "Molecular Subtype"[TW] OR "Molecular Subtypes"[TW] OR "Molecular Subtyping"[TW] OR "Molecular Classifier"[TW] OR "Molecular Classifiers"[TW] OR "Molecular Profile"[TW] OR "Molecular Profiles"[TW] OR "Molecular Profiling"[TW] OR "Genomic Profile"[TW] OR "Genomic Profiles"[TW] OR "Genomic Profiling"[TW] OR "Genomic Feature"[TW] OR "Genomic Features"[TW] OR "TCGA Classification"[TW] OR "TCGA Subtype"[TW] OR "TCGA Subtypes"[TW] OR "ProMisE"[TW] OR "Proactive Molecular Risk Classifier for Endometrial Cancer"[TW] OR "Mismatch Repair"[TW] OR "Mismatch Repair Deficiency"[TW] OR "Mismatch Repair Deficient"[TW] OR "MMR"[TW] OR "MMRd"[TW] OR "Microsatellite Instability"[TW] OR "Microsatellite Instability-High"[TW] OR "Microsatellite Instability High"[TW] OR "MSI"[TW] OR "MSI-H"[TW] OR "Tumor Mutational Burden"[TW] OR "Tumour Mutational Burden"[TW] OR "Tumor Mutation Burden"[TW] OR "Tumour Mutation Burden"[TW] OR "TMB"[TW] OR "POLE"[TW] OR "POLE Mutation"[TW] OR "POLE Mutations"[TW] OR "POLE-Mutated"[TW] OR "POLE Mutant"[TW] OR "POLEmut"[TW] OR "p53"[TW] OR "TP53"[TW] OR "p53 Abnormal"[TW] OR "p53-Abnormal"[TW] OR "p53abn"[TW] OR "TP53 Mutation"[TW] OR "TP53 Mutations"[TW] OR "Copy Number"[TW] OR "Copy-Number"[TW] OR "Copy Number High"[TW] OR "Copy-Number High"[TW] OR "Copy Number Low"[TW] OR "Copy-Number Low"[TW] OR "Copy Number Alteration"[TW] OR "Copy Number Alterations"[TW] OR "Copy Number Variation"[TW] OR "Copy Number Variations"[TW] OR "CNA"[TW] OR "CNV"[TW] OR "No Specific Molecular Profile"[TW] OR "No Specific Molecular Profile Subtype"[TW] OR "NSMP"[TW]) AND (2015:2026[pdat]) NOT ("Review"[pt] OR "Systematic Review"[pt] OR "Meta-Analysis"[pt] OR "Editorial"[pt] OR "Comment"[pt] OR "Letter"[pt] OR "Guideline"[pt] OR "Practice Guideline"[pt])

B. **Web of Science**

("endometrial cancer") and ("artificial intelligence" or "AI" OR "machine learning" or "deep learning" or "transfer learning" or "hierarchical learning" or "ensemble learning" or "multimodal deep learning" or "foundation model" or "foundation models") and ("Molecular Classification" or "Molecular Subtype" or "Molecular Classifier" or "Molecular Profile" or "Mismatch Repair" or "Microsatellite Instability" or "MSI" or "Tumor Mutational Burden" or "TMB" or "POLE" or "p53" or "TP53" or "Copy Number" or "No Specific Molecular Profile" or "NSMP")
